# Sociodemographic and health factors associated with genetic testing in Australia: insights from a cohort-based study of 45,061 participants

**DOI:** 10.1038/s41431-025-01816-x

**Published:** 2025-02-27

**Authors:** David E. Goldsbury, Yoon-Jung Kang, Catherine Tang, Hamzeh M. Tanha, Amelia K. Smit, Kate L. A. Dunlop, Lara Petelin, Preston Ngo, Harriet Hui, Nicola S. Meagher, Melissa A. Merritt, Marianne Weber, Anna DeFazio, Anne E. Cust, Karen Canfell, Julia Steinberg

**Affiliations:** 1 The Daffodil Centre, The University of Sydney, a joint venture with Cancer Council NSW, Sydney, NSW Australia; 2 The University of Sydney, Sydney, NSW Australia; 3 Melbourne School of Population & Global Health, University of Melbourne, Melbourne, VIC Australia; 4 Department of Gynaecological Oncology, Westmead Hospital, Sydney, NSW Australia; 5 The Westmead Institute for Medical Research, Sydney, NSW Australia; 6 School of Public Health, The University of Sydney, Sydney, NSW Australia

**Keywords:** Public health, Genetic testing

## Abstract

With increasing availability of genetic tests, it is important to consider differences in testing patterns between population subgroups. We examined self-reported genetic testing among 45,061 participants of the Australian population-based 45 and Up Study, testing for associations with sociodemographic and health characteristics (multivariable logistic regression). 9.2% of participants reported ever having genetic testing; 3.9% reported disease-related testing, 5.2% non-disease-related testing, 0.7% both disease-related and non-disease-related testing. Disease-related genetic testing was strongly associated with younger age, female sex, history of cancers and cardiovascular disease, and cancer family history. Disease-related testing was also strongly associated with higher education (university *versus* school certificate: adjusted OR [aOR] = 1.50 [95%CI:1.29–1.75]; certificate/diploma *versus* school certificate: aOR = 1.40 [95%CI:1.20–1.63]); there was suggestive evidence for association with higher household income ($AUD90,000+ *versus* <$AUD30,000: aOR = 1.22 [95%CI:1.02–1.46]), which strengthened when not adjusting for education (aOR = 1.34 [95%CI:1.13–1.60]). These results suggest further work on ensuring equitable access is needed to prevent potential health inequities.

## Introduction

Genetic and genomic testing (in the following, “genetic testing” for brevity) has considerable promise for precision health, with tests increasingly available for disease risk prediction, diagnosis, and treatment [1, 2], especially in cancer [3, 4]. Australia has universal healthcare (‘Medicare’), supplemented by private health insurance; however, re-imbursement for genetic testing is limited, with many tests covered by State/Territory Governments, private healthcare providers, and/or individuals [5]. Notably, disease-related direct-to-consumer (DTC) tests are increasingly available without a specialist referral, alongside non-disease-related DTC tests that can increase familiarity with genetics and uptake of future testing. To determine how genomics could support effective, efficient, and equitable healthcare, it is thus important to understand current patterns of genetic testing.

Australian studies from 2016 to 2017 [6, 7] reported that health literacy and socioeconomic advantage were associated with increased access to genetic tests (Supplementary Information p19). Since then, availability of genetic testing has increased substantially [8]. Here, we draw on more recent and larger-scale population-based data to investigate self-reported genetic testing (any, disease-related, and non-disease-related) and examine associations with sociodemographic and health characteristics (cancer and non-cancer conditions) in Australia.

## Materials and methods

### 45 and Up Study

The Sax Institute’s 45 and Up Study is a population-based cohort in New South Wales (NSW), Australia, with 267,357 participants aged 45+ years recruited in 2005–2009 [9, 10]. Briefly, potential participants were randomly sampled from the Services Australia Medicare enrolment database (1,395,174 invitations sent, ~19% participation rate). People aged 80+ years and rural/remote residents were oversampled. In 2020, questionnaires were sent to approximately one-third of the cohort (85,299 participants) as part of regular follow-up (52.8% response rate, details see Supplementary Information p3, Supplemental Fig. 1).

### Genetic testing

The 2020 follow-up questionnaire (paper-based or online) asked whether participants ever had any genetic testing (Yes; No; Don’t know/don’t want to say), and if so, what the genetic testing aimed to determine (multiple-choice question, see Supplementary Information p7). The questions were deliberately broad to avoid disclosure of testing with life insurance implications, without separating clinical and non-clinical settings. For subsequent analyses, we considered three genetic testing categories: “any testing” (ever had any genetic testing); “disease-related testing” (disease risk, diagnosis, or treatment); and “non-disease-related testing only” (genetic ancestry and/or diet-/fitness-related tests, but not tests related to disease risk, diagnosis or treatment).

### Participants’ characteristics

Participants’ sociodemographic and health characteristics were obtained from the 2020 or baseline questionnaire, including age, sex, education, household income, health insurance status, area-based socioeconomic status [11], accessibility/remoteness of place of residence [12], personal and family history of different diseases, and ever having children (details see Table 1, Supplementary Information p4). For the health characteristic of personal cancer history, participants’ invasive cancer diagnoses were ascertained from probabilistic linkage [13] to NSW Cancer Registry data (1994–2019; Table 1, Supplemental Table 1; registry data held by Cancer Institute NSW, linkage by the Centre for Health Record Linkage, http://www.cherel.org.au/).

**Table 1:** Characteristics of 45,061 participants included in the analysis.

Characteristics^a^	No. of participants	% of all participants
Age at 2020 follow-up (median age: 70 years; interquartile range: 64–76 years)		
56–59 years	3403	7.6%
60–69	19,079	42.3%
70–79	15,603	34.6%
80+	6976	15.5%
Sex		
Male	19,848	44.0%
Female	25,213	56.0%
Education: highest educational qualification, reported on individual level at cohort recruitment^b^		
No school certificate or other qualifications/School or intermediate certificate	10,377	23.0%
Higher school or leaving certificate	4010	8.9%
Trade/apprenticeship	3953	8.8%
Certificate/diploma	10,898	24.2%
University degree or higher	15,430	34.2%
Unknown/no response	393	0.9%
Household income: annual pre-tax income, reported on household level ($AUD)		
<$30,000	9649	21.4%
$30,000–<$50,000	7744	17.2%
$50,000–<$90,000	11,172	24.8%
$90,000+	9605	21.3%
Unknown/Prefer not to answer	6891	15.3%
Health insurance status		
Medicare only *(including those with no private health insurance, no healthcare concession card, and no Department of Veterans’ Affairs White or Gold Card)*	5049	11.2%
Healthcare concession card	6458	14.3%
Department of Veterans’ Affairs healthcare coverage (White or Gold card)	657	1.5%
Private health insurance (with/without extras)	32,897	73.0%
Area-based socioeconomic status: quintile of index of relative socioeconomic disadvantage, based on place of residence on area level [11]		
Most disadvantaged	7190	16.0%
Quintile 2	8696	19.3%
Quintile 3	8282	18.4%
Quintile 4	8278	18.4%
Least disadvantaged	10,447	23.2%
Missing	2168	4.8%
Accessibility/Remoteness of place of residence: based on place of residence on area level [19]		
Major cities	22,387	49.7%
Inner regional	16,176	35.9%
Outer regional	4405	9.8%
Remote/Very Remote	333	0.7%
Missing	1760	3.9%
Personal history of invasive cancer diagnosis in 1994–2019: based on NSW Cancer Registry linked data^c^		
Cancer diagnosis	7916	17.6%
No cancer diagnosis	37,145	82.4%
Detailed personal history of cancer diagnosis in 1994–2019: based on NSW Cancer Registry linked data, for *n* = 7916 participants with record of invasive cancer diagnosis^c^		
Breast cancer (ICD-10 code C50)	1717	3.8%
Colorectal cancer (ICD-10 code C18-20)	932	2.1%
Lung cancer (ICD-10 code C33-34)	133	0.3%
Melanoma (ICD-10 code C43)	1550	3.4%
Prostate cancer (ICD-10 code C61)	2376	5.3%
Other cancer (ICD-10 code C00-97, excluding C18-20, C33-34, C43, C50, and C61)	2013	4.5%
Personal history of other health conditions: based on self-report		
Cardiovascular disease (incl. heart failure, atrial fibrillation, blood clots, other heart disease and stroke)	11,497	25.5%
Diabetes (Type 1/Type 2 or unsure)	4731	10.5%
Family history of cancer: related to mother, father, and/or sibling(s), blood relatives only		
Any cancer	24,182	53.7%
Breast cancer	6721	14.9%
Colorectal cancer	7738	17.2%
Lung cancer	5608	12.4%
Melanoma	5908	13.1%
Ovarian cancer	1493	3.3%
Prostate cancer	6635	14.7%
Family history of non-cancer conditions: related to mother, father, and/or sibling(s), blood relatives only		
Heart disease	23,260	51.6%
Stroke	13,318	29.6%
Dementia /Alzheimer’s	12,292	27.3%
Diabetes	11,151	24.7%
Ever having children: based on number of children ever given birth to/fathered, reported at cohort recruitment^b^		
Yes *(1+ children given birth to/fathered)*	39,176	86.9%
No	5885	13.1%

All 45 and Up Study questionnaires and data books (including the baseline questionnaire and 2020 follow-up questionnaire) can be accessed from the Sax Institute (https://www.saxinstitute.org.au/solutions/45-and-up-study/use-the-45-and-up-study/data-and-technical-information, accessed 11 October 2024).

^a^Information was based on the 2020 follow-up questionnaire unless specified otherwise. For all characteristics based on questionnaire data, “missing” was included as a separate category in regression analyses.

^b^These characteristics were based on the baseline questionnaire [9, 10].

^c^Determined based on all records of invasive cancer (excluding keratinocyte/non-melanoma skin cancers) in the NSW Cancer Registry, including cancer type and year of diagnosis. ICD-10-codes are provided in parentheses. Due to the relatively small number of cases (*n* = 70), ovarian cancer was included in the “Other cancer” group.

### Statistical analyses

We reported the number and proportion of respondents for each genetic testing category, with exploratory analysis applying re-weighting for selected sociodemographic characteristics to Australian Census data (people aged 55+ years).

Multivariable logistic regression was used to test for associations between participants’ characteristics and genetic testing, separately for each of the three genetic testing categories (any, disease-related, or non-disease-related only). We calculated odds ratios (aOR) simultaneously adjusted for all characteristics shown in Table 1, and 95% confidence intervals (95%CI). To account for multiple testing (≤50 non-reference categories per analysis), we defined significance at *p* < 0.001 (Bonferroni-adjusted threshold). To indicate potential avenues for further work, we also reported associations at *p* < 0.05 as “suggestive evidence”.

Due to strong associations between genetic testing and both personal and family history of cancers, we further tested for associations specifically among participants with a previous invasive cancer diagnosis.

We performed several sensitivity analyses for the association tests: (1) for any genetic testing, excluding participants with “don’t know/don’t want to say” and missing responses (grouped with responses of no genetic testing in main analysis); (2) without adjustment for education, to examine associations between genetic testing and different socioeconomic status (SES) characteristics (due to correlation between education and SES); (3) excluding participants with personal or family history of cancer (to check for sex-specific cancers driving association between genetic testing and sex); (4) applying re-weighting to Australian Census data (exploratory only); and (5) stratified by sex.

Analyses used SAS v9.4 or R v4.3.1.

## Results

45,061 participants who completed the 2020 follow-up questionnaire could be included in the analysis (age at follow-up 56+ years, Table 1, Supplemental Fig. 1). Among all participants, 9.2% (95%CI:8.9–9.4%) reported ever having any genetic testing, 3.9% (3.7–4.1%) disease-related testing, 5.2% (5.0–5.4%) non-disease-related testing, and 0.7% (0.6–0.8%) both disease-related and non-disease-related testing (Supplemental Tables 2–3). Estimates were similar when re-weighting data to match the distribution of selected key characteristics to national or NSW data (absolute difference <0.6%, e.g. any genetic testing: 8.6–9.3%, Supplemental Table 4).

### Associations between genetic testing and participants’ characteristics

**Fig. 1:**
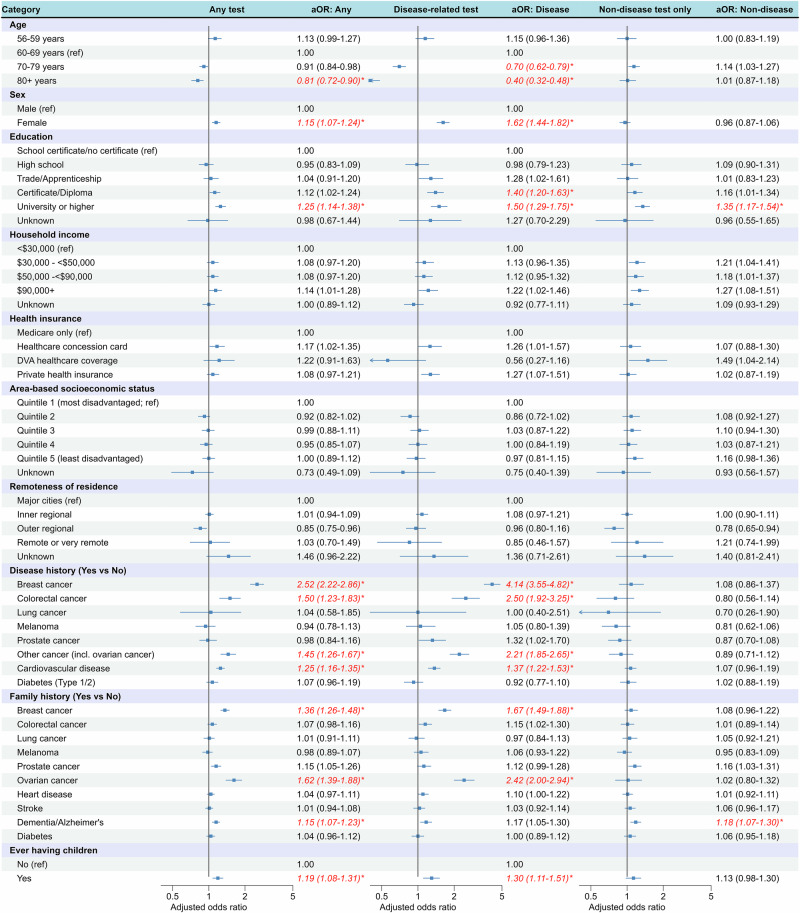
Associations between participants’ characteristics and any, disease-related, and non-disease related self-reported genetic testing (based on *n* = 45,061 participants of the 45 and Up Study followed up in 2020 who were included in the analysis). aOR: Odds ratio (OR)s adjusted for all characteristics shown here, alongside 95% confidence intervals in parentheses. Horizontal bars represent 95% confidence intervals; DVA: Department of Veterans’ Affairs. * Associations significant at *p* < 0.001 (Bonferroni-corrected threshold accounting for multiple testing). The reference category for both personal and family history of diseases was defined within each disease, i.e. estimates relate to participants with a specific disease compared to those without that specific disease, or to participants with family history of a specific disease to those without family history of that specific disease.

Ever having genetic testing was associated with age (80+ years: aOR = 0.81 versus 60–69 years) and female sex (aOR = 1.15 versus male; Fig. 1). There was a significant association with university education (aOR = 1.25 versus school certificate) and suggestive evidence (*p* < 0.05) for $AUD90,000+ household income (aOR = 1.14 versus <$AUD30,000), but no evidence for association with area-based SES or remoteness of residence. Significant associations were also observed with personal history of breast cancer, colorectal cancer and cardiovascular disease, family history of breast cancer, ovarian cancer and dementia/Alzheimer’s, and ever having children.

Disease-related testing showed similar association patterns, including stronger associations with age (70–79 years: aOR = 0.70; 80+ years: aOR = 0.40) and female sex (aOR = 1.62). Notably, we found stronger associations for several SES characteristics: significant associations for both certificate/diploma (aOR = 1.40) and university education (aOR = 1.50), suggestive evidence (*p* < 0.05) and a higher estimate for $AUD90,000+ household income (aOR = 1.22), and suggestive evidence for private health insurance (aOR = 1.27).

Reporting non-disease-related testing only was significantly associated with university education (aOR = 1.35) and family history of dementia/Alzheimer’s (aOR = 1.18; Fig. 1).

Results of analyses restricted to participants with a personal cancer history were similar to the main analysis (Supplementary Information p12). Disease-related testing was also significantly associated with younger age at diagnosis, more recent diagnosis periods, and metastatic/unknown spread of cancer at diagnosis (Supplemental Fig. 2).

### Sensitivity analyses

Excluding participants with “don’t know/don’t want to say” and missing responses to any genetic testing (5% of all *n* = 45,061) from the regression analysis had very little impact on the results.

Without adjustment for education, associations with higher household income increased (relative increase in aOR up to ~10%) and were statistically significant for $AUD90,000+ income (Supplementary Fig. 3). Associations with other characteristics did not change substantially. There was a similar pattern in this analysis restricted to participants with cancer, with aORs for disease-related testing and $AUD90,000+ household income increasing, though not statistically significant (Supplementary Fig. 4).

When the main association analyses were restricted to participants without any personal nor family history of cancer, the association between disease-related testing and sex was slightly attenuated (aOR = 1.47) but remained significant (Supplementary Fig. 5), suggesting testing related to sex-specific cancers is not the only contributing factor for this association between genetic testing and sex.

When re-weighting study data to the Australian population, association results were generally similar to the main analysis (Supplemental Table 5; Supplementary Information p17). Results from sex-stratified analyses were also largely similar, with most notable differences of stronger association between genetic testing (disease-related and non-disease-related) and university education among males than females, and family history of breast cancer associated with genetic testing (any and disease-related) among females only (Supplementary Table 6; Supplementary Information p18).

## Discussion

In this large-scale analysis of self-reported genetic testing among >45,000 Australians (age 56+ years) from a population-based cohort, 9.2% of participants reported ever having any genetic testing, among whom 42.4% reported disease-related testing and 56.3% non-disease-related testing, with 7.9% reporting both (see Supplementary information p20 for additional discussion). Re-weighted estimates to match the general population age 55+ were similar to the main estimates.

Self-reported genetic testing in our study was substantially lower than the 21.6% reported in a cross-sectional 2020 US survey [14], with the USA currently representing the largest genetic testing market. Our estimate was also lower than the 22.4% reported in the Australian Genioz study [7], which might be related to different participant demographics (56+ versus 18+ years; 56% versus 72% females) and/or recruitment (established cohort versus mix of strategies including social media; notably, 59% of Genioz study participants were undertaking/had university education, and 15% were working in life science/genomics, which likely contributed to the high prevalence of genetic testing).

Consistent with previous studies [6, 7, 14], we found strong associations between genetic testing and younger age and female sex (not explained by sex-specific cancers alone, with potential contributions of different health awareness and attitudes toward preventative care [15]).

We also found very strong associations between genetic testing and education. While previous Australian studies [6, 7] generally focused on university education only, we found a gradient across education levels. Compared to attaining at most a school certificate, odds ratio estimates for disease-related genetic testing were highest for university education, followed by certificate/diploma (both *p* < 0.001), then trade/apprenticeship qualifications (*p* < 0.05). Notably, we found evidence for stronger association between disease-related genetic testing and university education among males than females, which could be of interest for future investigation. Generally, associations with education could be related to increased health literacy and/or higher income facilitating out-of-pocket expenses for non-reimbursed tests (latter also supported by the increased and significant association with the highest household income when not adjusting for education). Out-of-pocket expenses for genetic testing are also highly relevant for DTC tests, with potential for health inequities discussed further in Supplementary Information p21.

The strong associations between genetic testing and personal and family history of several cancers were consistent with expectations based on current Australian genetic testing guidelines [16, 17] and increased use of genetic testing for targeted treatment [18] (germline and somatic tests were not separated in the self-report). We found a significant association between CVD and genetic testing, consistent with increasing availability of genetic tests for e.g., inherited cardiomyopathy and inherited hypercholesterolemia [19].

As a study limitation, the cohort was not representative of the general population (e.g., due to older age, higher education and socioeconomic advantage); nonetheless, previous work suggests within-cohort associations are expected to mirror population relationships [20]. Self-reported genetic testing is subject to recall bias, which could differ by age and/or education. We could not distinguish whether genetic testing occurred through health professionals. Notable strengths of this study include the very large sample, inclusion of a very broad range of participants’ characteristics, data linkage to cancer registry, and rigorous statistical analysis.

In conclusion, our results provide insights on genetic testing patterns in Australia as an example of a high-income country, and re-enforce the need for further work to ensure equitable access to current and future genomic technologies, covering both educational and financial considerations in depth.

## Supplementary information

Appendix A

Appendix B

Appendix C

## Data Availability

This study uses third-party data not owned or collected by the authors, with on-provision by authors not permitted by the relevant data custodians (Sax Institute, Cancer Institute NSW), as it would compromise the participants’ confidentiality and privacy. However, the data are available from the data custodians for approved research projects - data access enquiries can be made to the Sax Institute (see https://www.saxinstitute.org.au/our-work/45-upstudy/governance/ for details). Other researchers would be able to access these data using the same process followed by the authors.
