# Supplementary material for: Sociodemographic and health factors associated with genetic testing in Australia: insights from a cohort-based study of 45,061 participants
Source: Eur J Hum Genet. 2025 Feb 27;33(6):819–24. doi: 10.1038/s41431-025-01816-x (PMC12185706; doi:10.1038/s41431-025-01816-x)
Supplement: Supplementary file 1 — Appendix A [file 41431_2025_1816_MOESM1_ESM.docx]

**Sociodemographic and health factors associated with genetic testing in Australia: insights from a cohort-based study of 45,061 participants**

**Supplementary Information**

Table of Contents

[1. Study participants and categorisation of responses to questions on genetic testing 3](#_Toc189141392)

[1) Participant inclusion 3](#_Toc189141393)

[2) Participants’ characteristics 4](#_Toc189141394)

[3) Questionnaire items related to genetic testing, from the 2020 follow-up of the 45 and Up Study 7](#_Toc189141395)

[2. Uptake of self-reported genetic testing 8](#_Toc189141396)

[3. Associations between genetic testing and participants’ characteristics, among participants with a history of cancer 12](#_Toc189141397)

[4. Association with income, without adjusting for educational attainment 14](#_Toc189141398)

[5. Association with sex, after removing participants with personal or family history of cancer 16](#_Toc189141399)

[6. Associations between genetic testing and participants’ characteristics, applying re-weighting to Australian Census data (people aged 55+ years) 17](#_Toc189141400)

[7. Associations between genetic testing and participants’ characteristics, stratified by sex 18](#_Toc189141401)

[8. Previous studies of genetic testing and associations with sociodemographic characteristics 19](#_Toc189141402)

[9. Overlap in disease-related and non-disease-related genetic testing 20](#_Toc189141403)

[10. Availability of disease-related DTC tests in Australia and considerations related to health disparities 21](#_Toc189141404)

[11. References 22](#_Toc189141405)

List of tables

[Supplemental Table 1. Characteristics of 7,916 participants with a history of cancer included in the analysis 5](#_Toc189141383)

[Supplemental Table 2. Self-reported genetic testing of 45 and Up Study participants included in the analysis (n=45,061) 8](#_Toc189141384)

[Supplemental Table 3. Self-reported genetic testing of 45 and Up Study participants included in the analysis (n=45,061), by participants’ characteristics 9](#_Toc189141385)

[Supplemental Table 4. Self-reported genetic testing of 45 and Up Study participants included in the analysis (n=45,061), after re-weighting to match selected sociodemographic characteristics to Australian Census population data 11](#_Toc189141386)

List of figures

[Supplemental Fig. 1. Participant selection and data sources 3](#_Toc189141378)

[Supplemental Fig. 2 Associations between participants' characteristics and self-reported genetic testing among participants with a history of cancer (based on n=7,916 participants of the 45 and Up Study followed up in 2020 who were included in the analysis) 13](#_Toc189141379)

[Supplemental Fig. 3 Sensitivity analysis: associations between participants' characteristics and self-reported genetic testing, without adjustment for educational attainment (based on n=45,061 participants of the 45 and Up Study followed up in 2020 who were included in the analysis) 14](#_Toc189141380)

[Supplemental Fig. 4 Sensitivity analysis: associations between participants' characteristics and self-reported genetic testing among participants with a history of cancer, without adjustment for educational attainment 15](#_Toc189141381)

[Supplemental Fig. 5 Sensitivity analysis: association between participants’ characteristics and genetic testing among participants with no personal and no family history of cancer 16](#_Toc189141382)

# 1. Study participants and categorisation of responses to questions on genetic testing

## 1) Participant inclusion

Detailed information on the Sax Institute’s 45 and Up Study recruitment is available in prior studies describing the cohort [1, 2]. Briefly, potential participants were randomly sampled from the Services Australia Medicare enrolment database if they had received medical care within the previous 2 years, oversampling people aged 80+ years and rural/remote residents. A total of 1,395,174 invitations were mailed to the NSW residents aged 45+ years in 2005-2009 and 267,357 participants enrolled (~19% response rate, representing 11% of NSW residents aged 45+ years).

45 and Up Study participants are followed up roughly every 5 years: Baseline collection (2006–2009); Wave 2 (2012–2015); Wave 3 (2018–2020). At the start of follow-up wave, all remaining contactable participants who have not died and have not withdrawn from the study are split into recruitment groups, or “phases” of that wave. For Wave 3, there were three phases: Phase 1 (2018); Phase 2 (2019); Phase 3 (2020). In general, the split into “phases” is based on the baseline recruitment date, in order to achieve a gap of approximately 5 years between surveys.

In 2020, questionnaires were sent to approximately one-third of the cohort (85,299 participants) as part of regular follow-up (Wave 3, Phase 3). Of the 45,078 participants who completed the survey (52.8% response rate), 45,061 participants could be included in the analysis (excluding those whose data were unavailable, e.g. due to withdrawal after questionnaire completion, see Supplemental Fig. 1). The 52.8% response rate to the 2020 follow-up questionnaires compares favourably to response rates for follow-up questionnaires in 2018 and 2019 (38.8% and 46.8%, respectively) [3].

In our study, a personal history of cancer was determined from linkage to the NSW Cancer Registry, based on data for 1994-2019. Thus, personal history of cancer would not have been identified for those diagnosed prior to 1994, or in the first months of 2020 prior to questionnaire completion.

Supplemental Fig. 1. Participant selection and data sources

## 2) Participants’ characteristics

The 45 and Up Study questionnaires included standard questions on date of birth, as well as questions on education and household income, similar to questions used in the Australian Census. It also included questions on personal history of different diseases as well as family history of cancers, heart disease, stroke, diabetes, dementia/Alzheimer’s and diabetes (in a check-box/multiple response format), similar to questionnaires used for accessing family history of diseases that have been shown to have a strong face and content validity [4, 5].

Participants’ sociodemographic and health characteristics were obtained from the 2020 follow-up questionnaire or baseline questionnaire as follows (see also Table 1). Sociodemographic characteristics included

- age;
- sex;
- education (self-reported on individual level at baseline, with categories: no school certificate; higher school or leaving certificate; trade/apprenticeship; certificate/diploma/ university degree or higher; unknown/no response);
- household income (self-reported on household level at 2020 follow-up, with categories <$30K; $30K-<$50K; $50K-<$90K; $90K+; unknown/prefer not to answer),
- health insurance status (reported on individual level at 2020 follow-up);
- area-based socioeconomic status, which was the Socio-Economic Indexes for Areas (SEIFA) Index of Relative Socio-economic Disadvantage (IRSD) [6]. The IRSD combines Census data on 15 area-level indicators of disadvantage (e.g. % of people in households with low income, % of people in labour force unemployed) and ranks each area relative to other areas. IRSD is one of four SEIFA indexes and was provided as part of the 45 and Up Study data based on geo-coded residence area at 2020 follow-up; and
- accessibility/remoteness of place of residence, which was based on the Accessibility/Remoteness Index of Australia (ARIA+) area-level indicator [7]. ARIA+ is a relative measure of remoteness across Australia and was provided as part of the 45 and Up Study data based on geo-coded residence area at 2020 follow-up.

Health characteristics include personal and family history of cancer, personal history of cardiovascular disease and diabetes, and family history of heart disease, stroke, dementia/Alzheimer’s and diabetes. The 2020 follow-up questionnaire did not ask participants whether they had a personal history of dementia/Alzheimer’s, thus these conditions were included as categories for family history only. We also considered whether participants had ever had children, as individuals with children may be more likely to pursue disease-related testing to inform prevention and screening options for their children.

To examine personal cancer history, invasive cancer diagnoses (excluding keratinocyte/non-melanoma skin cancers) were ascertained from linked NSW Cancer Registry data (1994-2019), including cancer type, diagnosis year, spread of disease and age at diagnosis [8]. Diagnoses of keratinocyte/non-melanoma skin cancers were not included in this study as these cancers are not recorded within the NSW Cancer Registry data. Analogous population-wide registries for other conditions (e.g. CVD, diabetes) do not exist in Australia or were not available for this study. Information from alternative administrative data is not as comprehensive; for example, hospital records in the NSW Admitted Patient Data Collection (APDC) can only be used to identify conditions related to hospital admissions. Thus, we relied on self-report for personal history and family history of non-cancer conditions.

Supplemental Table 1. Characteristics of 7,916 participants with a history of cancer included in the analysis

|  | **Participants with a history of cancer (n=7,916)** | |
| --- | --- | --- |
| **Characteristics ^a^** | **No. of participants** | **% of participants** |
| **Age** **at 2020 follow-up** (**median age: 74 years; interquartile range: 67-79 years)** | | |
| 56-59 years | 254 | 3.2% |
| 60-69 | 2,317 | 29.3% |
| 70-79 | 3,370 | 42.6% |
| 80+ | 1,975 | 24.9% |
| **Sex** |  |  |
| Male | 4,260 | 53.8% |
| Female | 3,656 | 46.2% |
| **Education**: highest education qualification reported on individual level at cohort recruitment ^b^ | | |
| No school certificate or other qualifications/School or intermediate certificate | 1,959 | 24.7% |
| Higher school or leaving certificate | 698 | 8.8% |
| Trade/apprenticeship | 788 | 10.0% |
| Certificate/diploma | 1,824 | 23.0% |
| University degree or higher | 2,584 | 32.6% |
| Unknown/no response | 63 | 0.8% |
| **Household income**: annual pre-tax income reported on household level ($AUD) | | |
| <$30,000 | 1,976 | 25.0% |
| $30,000 - <$50,000 | 1,485 | 18.8% |
| $50,000 - <$90,000 | 1,870 | 23.6% |
| $90,000+ | 1,386 | 17.5% |
| Unknown/Prefer not to answer | 1,199 | 15.1% |
| **Health insurance status** | | |
| Medicare only *(including those with no private health insurance, no healthcare concession card, and no Department of Veterans' Affairs White or Gold Card)* | 694 | 8.8% |
| Healthcare concession card | 1,252 | 15.8% |
| Department of Veterans' Affairs healthcare coverage (White or Gold card) | 146 | 1.8% |
| Private health insurance (with/without extras) | 5,824 | 73.6% |
| **Area-based socioeconomic status:** quintile of index of relative socioeconomic disadvantage, based on place of residence on area level [6] | | |
| Most disadvantaged | 1,318 | 16.6% |
| Quintile 2 | 1,521 | 19.2% |
| Quintile 3 | 1,452 | 18.3% |
| Quintile 4 | 1,483 | 18.7% |
| Least disadvantaged | 1,864 | 23.5% |
| Missing | 278 | 3.5% |
| **Accessibility/Remoteness of place of residence**: based on place of residence on area level [7] | | |
| Major cities | 4,021 | 50.8% |
| Inner regional | 2,907 | 36.7% |
| Outer regional/ Remote/Very Remote | 782 | 9.9% |
| Missing | 206 | 2.6% |
| **Personal history of invasive cancer diagnosis in 1994-2019:** based on the earliest NSW Cancer Registry linked record ^c^ | | |
| Breast cancer (ICD-10 code C50) | 1,637 | 20.7% |
| Colorectal cancer (ICD-10 code C18-20) | 820 | 10.4% |
| Lung cancer (ICD-10 code C33-34) | 97 | 1.2% |
| Melanoma (ICD-10 code C43) | 1,383 | 17.5% |
| Prostate cancer (ICD-10 code C61) | 2,203 | 27.8% |
| Other cancer (ICD-10 code C00-97, excluding C18-20, C33-34, C43, C50, and C61) | 1,776 | 22.4% |
| **Cancer diagnosis period** | | |
| Pre-2005 | 2,491 | 31.5% |
| 2005-2009 | 2,020 | 25.5% |
| 2009-2014 | 1,645 | 20.8% |
| 2015-2019 | 1,760 | 22.2% |
| **Cancer spread at diagnosis** | | |
| Localised | 4,672 | 59.0% |
| Regional | 1,529 | 19.3% |
| Metastatic/unknown | 1,715 | 21.7% |
| **Age at cancer diagnosis** | | |
| <60 years | 2,770 | 35.0% |
| 60-69 years | 3,003 | 37.9% |
| 70-79 years | 1,745 | 22.0% |
| 80+ years | 398 | 5.0% |
| **Personal history of other health conditions**: based on self-report | | |
| Cardiovascular disease (including heart failure, atrial fibrillation, blood clots, other heart disease and stroke) | 2,129 | 26.9% |
| Diabetes (Type 1/Type 2 or unsure) | 1,038 | 13.1% |
| **Family history of cancer**: related to mother, father, and/or sibling(s), blood relatives only | | |
| Breast cancer | 1,382 | 17.5% |
| Colorectal cancer | 1,476 | 18.6% |
| Lung cancer | 1,005 | 12.7% |
| Melanoma | 1,083 | 13.7% |
| Ovarian cancer | 1,405 | 17.7% |
| Prostate cancer | 280 | 3.5% |
| **Family history of other conditions**: related to mother, father, and/or sibling(s), blood relatives only | | |
| Heart disease | 4,135 | 52.2% |
| Stroke | 2,335 | 29.5% |
| Dementia /Alzheimer's | 1,973 | 24.9% |
| Diabetes | 1,849 | 23.4% |
| **Ever having children**: based on number of children given birth to/fathered, reported at cohort recruitment **^b^** | | |
| Yes *(1+ children given birth to/fathered)* | 6,910 | 87.3% |
| No | 1,006 | 12.7% |

^a^ Information was based on the 2020 follow-up questionnaire unless specified otherwise. For all characteristics based on questionnaire data, “missing” was included as a separate category in regression analyses.

^b^ These characteristics were based on the baseline questionnaire [1, 2].

^c^ Determined based on the first record of invasive cancer (excluding keratinocyte/non-melanoma skin cancers) in the NSW Cancer Registry, including cancer type, year of diagnosis, age at diagnosis, and summary spread of disease at diagnosis. ICD-10-codes are provided in parentheses. Due to the relatively small number of cases (n=70), ovarian cancer was included in the "Other cancer" group.

**Note**: All 45 and Up Study questionnaires and data books (including the baseline questionnaire and 2020 follow-up questionnaire) can be accessed from the Sax Institute (<https://www.saxinstitute.org.au/solutions/45-and-up-study/use-the-45-and-up-study/data-and-technical-information>).

## 3) Questionnaire items related to genetic testing, from the 2020 follow-up of the 45 and Up Study

The question on self-reported genetic testing (“have you ever had genetic testing”) was phrased as in the Genioz study, which reported personal genetic testing uptake in Australia in 2016-2017 and used a modified Delphi technique for face validity and refining question inclusion [9]. Response options on the types of tests were condensed for brevity, following a similar 2-stage approach as the Genioz study, with review by researchers as well as the Sax Institute’s team responsible for the 45 and Up Study.

Questions related to genetic testing were included in Wave 3, Phase 3 (2020) only. The 2020 follow-up questionnaire asked whether participants ever had genetic testing:

*QB79. “Have you ever had genetic testing?”* with three response options:

- *Yes;*
- *No;*
- *Don’t know/don’t want to say.*

If participants selected “Yes” to QB79, they were guided to the next question:

*QB80. “What did the genetic testing aim to determine? (shade all that apply)*” with four response options:

- *disease risk, diagnosis or treatment;*
- *my ancestry;*
- *other (e.g. targeting diet or fitness);*
- *don’t know/don’t want to say.*

For the analyses in this study, we categorised the above responses into three genetic testing categories:

1. **any genetic testing** (if selected “Yes” for QB79. “Have you ever had genetic testing?”);
2. **disease-related testing** (if selected “Yes” for QB79 and “disease risk, diagnosis or treatment” for QB80); and
3. **non-disease-related testing only** (if selected “Yes” for QB79; and for QB80, selected “my ancestry” and/or “other [e.g. targeting diet or fitness)”, but not “disease risk, diagnosis or management”).

Supplemental Table 2 shows the numbers of participants assigned to each of these categories: n=4,124 for any genetic testing, n=1,748 for disease-related testing, and n=1,998 for non-disease-related testing only. The disease-related testing and non-disease-related testing categories did not include n=378 participants who did not specify what the genetic testing aimed to determine.

All 45 and Up Study questionnaires and data books (including the baseline questionnaire and 2020 follow-up questionnaire) can be accessed from the Sax Institute (<https://www.saxinstitute.org.au/solutions/45-and-up-study/use-the-45-and-up-study/data-and-technical-information>).

# 2. Uptake of self-reported genetic testing

Supplemental Table 2. Self-reported genetic testing of 45 and Up Study participants included in the analysis (n=45,061)

|  | **No. of participants** | **% (95% CI) ^b^ of all participants (N=45,061)** | **% (95% CI) ^b^ of those tested (n=4,124)** |
| --- | --- | --- | --- |
| ***“Have you ever had genetic testing?”* ^a^** | | | |
| Yes | 4,124 | 9.2% (8.9-9.4%) | 100% |
| No | 38,691 | 85.9% (85.5-86.2%) | - |
| Don't know /don't want to say | 1,567 | 3.5% (3.3-3.6%) | - |
| Missing | 679 | 1.5% (1.4-1.6%) | - |
| ***If reported ever had genetic testing, “What did the genetic testing aim to determine?” ^c^*** | | | |
| Any genetic testing | 4,124 | 9.2% (8.9-9.4%) | 100% |
| Disease-related testing (‘disease risk, diagnosis or treatment’) | 1,748 | 3.9% (3.7-4.1%) | 42.4% (40.9-43.9%) |
| Non-disease-related testing (‘my ancestry’; ‘other [e.g. targeting diet or fitness]’) | 2,323 | 5.2% (5.0-5.4%) | 56.3% (54.8-57.8%) |
| *Non-disease-related testing only* | *1,998* | *4.4%* (4.2-4.6%) | *48.4% (46.9-50.0%)* |
| *Disease-related testing & non-disease-related testing* | *325* | *0.7%* (0.6-0.8%) | *7.9% (7.1-8.7%)* |
| No aims specified for test (don’t know/don’t want to say) | 378 | 0.8% (0.8-0.9%) | 9.2% (8.3-10.0%) |

95% CI: 95% confidence interval.

^a^ Note that questions were intentionally broad to avoid potential disclosure/insurance implications. Responses indicating "don't know/don't want to say" as well as missing responses were considered as not being tested for subsequent association analyses.

^b^ Number and proportion of respondents with each genetic testing response, with 95% confidence intervals (95%CIs) calculated using the normal approximation (as $P\pm1.96\sqrt{P*(1-P)/N}$ for the proportion *P* among *N* participants).

^c^ Multiple selection possible among “disease risk, diagnosis or treatment”; “my ancestry”; “other (e.g. targeting diet or fitness)”; “don’t know/don’t want to say”.

Supplemental Table 3. Self-reported genetic testing of 45 and Up Study participants included in the analysis (n=45,061), by participants’ characteristics

|  | **All participants (N=45,061)** | | **Self-reported genetic testing (row %)** | | |
| --- | --- | --- | --- | --- | --- |
| **Characteristics ^a^** | **No. of participants** | **% of all participants** | **Any genetic testing**  **(n=4,124)** | **Disease-related testing**  **(n=1,748)** | **Non-disease testing only**  **(n=1,998)** |
| **Age** **at 2020 follow-up** (**median age: 70 years; interquartile range: 67-76 years)** | | | | | |
| 56-59 years | 3,403 | 7.6% | 10.3% | 5.1% | 4.4% |
| 60-69 years | 19,079 | 42.3% | 9.6% | 4.7% | 4.4% |
| 70-79 years | 15,603 | 34.6% | 8.9% | 3.5% | 4.7% |
| 80+ years | 6,976 | 15.5% | 7.9% | 2.0% | 3.9% |
| **Sex** | | | | | |
| Male | 19,848 | 44.0% | 8.2% | 2.7% | 4.6% |
| Female | 25,213 | 56.0% | 9.9% | 4.8% | 4.3% |
| **Education**: highest education qualification reported on individual level at cohort recruitment ^b^ | | | | | |
| No school certificate or other qualifications/School or intermediate certificate | 10,377 | 23.0% | 8.3% | 2.9% | 3.7% |
| Higher school or leaving certificate | 4,010 | 8.9% | 7.8% | 2.9% | 4.1% |
| Trade/apprenticeship | 3,953 | 8.8% | 7.8% | 2.9% | 3.8% |
| Certificate/diploma | 10,898 | 24.2% | 9.3% | 4.3% | 4.4% |
| University degree or higher | 15,430 | 34.2% | 10.4% | 4.7% | 5.2% |
| Unknown/no response | 393 | 0.9% | 7.6% | 3.1% | 3.6% |
| **Household income**: annual pre-tax income reported on household level ($AUD) | | | | | |
| <$30,000 | 9,649 | 21.4% | 8.4% | 3.1% | 3.6% |
| $30,000 - <$50,000 | 7,744 | 17.2% | 9.0% | 3.7% | 4.5% |
| $50,000 - <$90,000 | 11,172 | 24.8% | 9.3% | 4.1% | 4.6% |
| $90,000+ | 9,605 | 21.3% | 10.2% | 4.8% | 5.1% |
| Unknown/Prefer not to answer | 6,891 | 15.3% | 8.7% | 3.5% | 4.1% |
| **Health insurance status** | | | | | |
| Medicare only *(including those with no private health insurance, no healthcare concession card, and no Department of Veterans' Affairs White or Gold Card)* | 5,049 | 11.2% | 8.1% | 3.1% | 4.0% |
| Healthcare concession card | 6,458 | 14.3% | 9.0% | 3.3% | 4.0% |
| Department of Veterans' Affairs healthcare coverage (White or Gold card) | 657 | 1.5% | 9.0% | 1.2% | 5.9% |
| Private health insurance (with/without extras) | 32,897 | 73.0% | 9.3% | 4.2% | 4.6% |
| **Area-based socioeconomic status:** quintile of index of relative socioeconomic disadvantage, based on place of residence on area level [6] | | | | | |
| Most disadvantaged | 7,190 | 16.0% | 9.0% | 3.6% | 3.8% |
| Quintile 2 | 8,696 | 19.3% | 8.4% | 3.3% | 4.2% |
| Quintile 3 | 8,282 | 18.4% | 9.2% | 4.1% | 4.4% |
| Quintile 4 | 8,278 | 18.4% | 9.1% | 4.2% | 4.3% |
| Least disadvantaged | 10,447 | 23.2% | 9.8% | 4.1% | 5.1% |
| Missing | 2,168 | 4.8% | 9.4% | 3.7% | 5.2% |
| **Accessibility/Remoteness of place of residence**: based on place of residence on area level [7] | | | | | |
| Major cities | 22,387 | 49.7% | 9.3% | 3.9% | 4.6% |
| Inner regional | 16,176 | 35.9% | 9.2% | 4.0% | 4.4% |
| Outer regional | 4,405 | 9.8% | 7.7% | 3.5% | 3.4% |
| Remote/Very Remote | 333 | 0.7% | 9.3% | 3.3% | 5.1% |
| Missing | 1,760 | 3.9% | 10.0% | 4.0% | 5.5% |
| **Personal history of invasive cancer diagnosis in 1994-2019**: based on NSW Cancer Registry linked data ^c^ | | | | | |
| Cancer diagnosis | 7,916 | 17.6% | 12.0% | 6.8% | 4.1% |
| No cancer diagnosis | 37,145 | 82.4% | 8.5% | 3.3% | 4.5% |
| **Detailed personal history of cancer diagnosis in 1994-2019 (from NSW Cancer Registry linked data, n=7,916) ^c^** | | | | | |
| Breast cancer (ICD-10 code C50) | 1,717 | 3.8% | 20.4% | 14.8% | 4.7% |
| Colorectal cancer (ICD-10 code C18-20) | 932 | 2.1% | 12.4% | 7.3% | 3.0% |
| Lung cancer (ICD-10 code C33-34) | 133 | 0.3% | 9.8% | *not reported due to cell size* | *not reported due to cell size* |
| Melanoma (ICD-10 code C43) | 1,550 | 3.4% | 8.4% | 3.7% | 3.6% |
| Prostate cancer (ICD-10 code C61) | 2,376 | 5.3% | 8.0% | 3.1% | 4.1% |
| Other cancer (ICD-10 code C00-97, excluding C18-20, C33-34, C43, C50, and C61) | 2,013 | 4.5% | 12.5% | 7.4% | 3.9% |
| **Personal history of other health conditions**: based on self-report | | | | | |
| Cardiovascular disease (including heart failure, atrial fibrillation, blood clots, other heart disease and stroke) | 11,497 | 25.5% | 10.2% | 3.9% | 4.6% |
| Diabetes (Type 1/Type 2 or unsure) | 4,731 | 10.5% | 9.4% | 3.2% | 4.4% |
| **Family history of cancer**: related to mother, father, and/or sibling(s), blood relatives only | | | | | |
| Any cancer | 24,182 | 53.7% | 9.9% | 4.6% | 4.5% |
| Breast cancer | 6,721 | 14.9% | 12.2% | 6.5% | 4.7% |
| Colorectal cancer | 7,738 | 17.2% | 9.9% | 4.6% | 4.5% |
| Lung cancer | 5,608 | 12.4% | 9.2% | 3.8% | 4.5% |
| Melanoma | 5,908 | 13.1% | 9.5% | 4.7% | 4.4% |
| Ovarian cancer | 1,493 | 3.3% | 14.0% | 8.6% | 4.5% |
| Prostate cancer | 6,635 | 14.7% | 10.4% | 4.6% | 5.0% |
| **Family history of other conditions**: related to mother, father, and/or sibling(s), blood relatives only | | | | | |
| Heart disease | 23,260 | 51.6% | 9.5% | 4.2% | 4.5% |
| Stroke | 13,318 | 29.6% | 9.3% | 4.0% | 4.7% |
| Dementia /Alzheimer's | 12,292 | 27.3% | 10.1% | 4.4% | 5.0% |
| Diabetes | 11,151 | 24.7% | 9.7% | 4.1% | 4.6% |
| **Ever having children**: based on number of children given birth to/fathered, reported at cohort recruitment ^b^ | | | | | |
| Yes *(1+ children given birth to/fathered)* | 39,176 | 86.9% | 9.3% | 4.0% | 4.5% |
| No | 5,885 | 13.1% | 8.2% | 3.4% | 4.1% |

^a^ Information was based on the 2020 follow-up questionnaire unless specified otherwise. For all characteristics based on questionnaire data, “missing” was included as a separate category in regression analyses.

^b^ These characteristics were based on the baseline questionnaire [1, 2].

^c^ Determined based on the most recent record of invasive cancer (excluding keratinocyte/non-melanoma skin cancers) in the NSW Cancer Registry, including cancer type and year of diagnosis. ICD-10-codes are provided in parentheses. Due to the relatively small number of cases (n=70), ovarian cancer was included in the "Other cancer" group.

**Note**: All 45 and Up Study questionnaires and data books (including the baseline questionnaire and 2020 follow-up questionnaire) can be accessed from the Sax Institute (<https://www.saxinstitute.org.au/solutions/45-and-up-study/use-the-45-and-up-study/data-and-technical-information>).

Supplemental Table 4. Self-reported genetic testing of 45 and Up Study participants included in the analysis (n=45,061), after re-weighting to match selected sociodemographic characteristics to Australian Census population data

|  | **Unweighted % (95% CI) of all participants** | **Re-weighted estimates, based on univariate matching of sociodemographic characteristics to those of the Australian population (2021 Census data) ^a^ Weighting based on:** | | | | | |
| --- | --- | --- | --- | --- | --- | --- | --- |
| **Responses** |  | **Age** | **Sex** | **Highest educational attainment** | **Household income** | **Area-based socioeconomic status** | **Accessibility/remoteness of place of residence** |
| ***Australian residents age 55+ years*** | | | | | | | |
| Any genetic testing | 9.2% (8.9-9.4%) | 9.3% | 9.1% | 8.6% | 8.9% | 9.1% | 9.2% |
| Disease-related testing | 3.9% (3.7-4.1%) | 4.0% | 3.8% | 3.5% | 3.6% | 3.9% | 3.9% |
| Non-disease-related testing only | 4.4% (4.2-4.6%) | 4.4% | 4.4% | 4.1% | 4.2% | 4.3% | 4.4% |
| ***New South Wales residents age 55+ years*** | | | | | | | |
| Any genetic testing | 9.2% (8.9-9.4%) | 9.3% | 9.1% | 8.7% | 8.9% | 9.1% | 9.2% |
| Disease-related testing | 3.9% (3.7-4.1%) | 4.0% | 3.8% | 3.5% | 3.6% | 3.9% | 3.9% |
| Non-disease-related testing only | 4.4% (4.2-4.6%) | 4.4% | 4.4% | 4.1% | 4.2% | 4.4% | 4.5% |

95% CI: 95% confidence interval.

^a^ Re-weighted estimates were obtained as $\sum_{i} r_{i}*p_{i}/c_{i}$, where for a given category $i$ of a characteristic (e.g. university education), $r_{i}$ is the proportion of 45 and Up Study participants with self-reported genetic testing among those with that category (e.g. disease-related genetic testing among those with university education), $p_{i}$ is the frequency of that category among the general population, and $c_{i}$ is the frequency of that category among the cohort participants included in the study. Distributions of the characteristics shown here were based on the Census 2021 data [10] among Australian residents aged 55+ years only, matching to the age groups in the cohort at the 2020 follow-up. Separately, we considered the distributions for among New South Wales residents aged 55+ years only, also using the Census data. The categories of characteristics were aligned with 45 and Up Study baseline questionnaire categories as in [11]. Due to data limitations, the annual household income categories from Census 2021 data were slightly different to the categories in this study, i.e. cohort categories of <$30k, $30k to <$50k, $50k to <$90k, and $90k were re-weighted to Census 2021 categories of <$26.02k, $26.7-52.09k, $52.14-91.25k, and >$91.25k per annum, respectively.

# 3. Associations between genetic testing and participants’ characteristics, among participants with a history of cancer

Due to the strong associations between genetic testing and both personal and family history of cancers, we further analysed characteristics associated with genetic testing among those with a previous cancer diagnosis (Supplemental Fig. 2).

Disease-related genetic testing showed similar association patterns to the main analysis of all participants (noting that as per data availability, disease-related testing here refers to testing for any disease, not necessarily related to cancer). We found strong association between disease-related genetic testing and female sex (aOR=1.75). While the sample size and thus power was reduced in this analysis compared to all participants, there was consistent suggestive evidence for association with university education (aOR=1.39 vs. school certificate; p<0.05) and private health insurance (aOR=1.54 vs. Medicare only; p<0.05). There remained no evidence for association with area-based SES or remoteness of residence.

Considering cancer-related characteristics, we found strong associations with breast cancer (aOR=2.82), more recent diagnosis periods (aOR=1.99 for diagnosis 2015-2019 vs. pre-2015), and metastatic/unknown spread of cancer at diagnosis (aOR=1.57 vs. localised). We also found a strong association with and younger age at diagnosis (aOR=2.16 for <60 vs. 60-69 years at cancer diagnosis; with a consistent trend for older age groups, see Supplemental Fig. 2). As in the main analysis of all participants, we found strong associations with family history of breast cancer (aOR=2.44) and ovarian cancer (aOR=2.67).

The focus of the analysis restricted to participants with cancer was primarily on disease-related testing; for completeness, association results for any genetic testing and non-disease-related testing only are also shown in Supplemental Fig. 2 (showing similar patterns to the analysis of all participants).

Supplemental Fig. 2 Associations between participants' characteristics and self-reported genetic testing among participants with a history of cancer (based on n=7,916 participants of the 45 and Up Study followed up in 2020 who were included in the analysis)


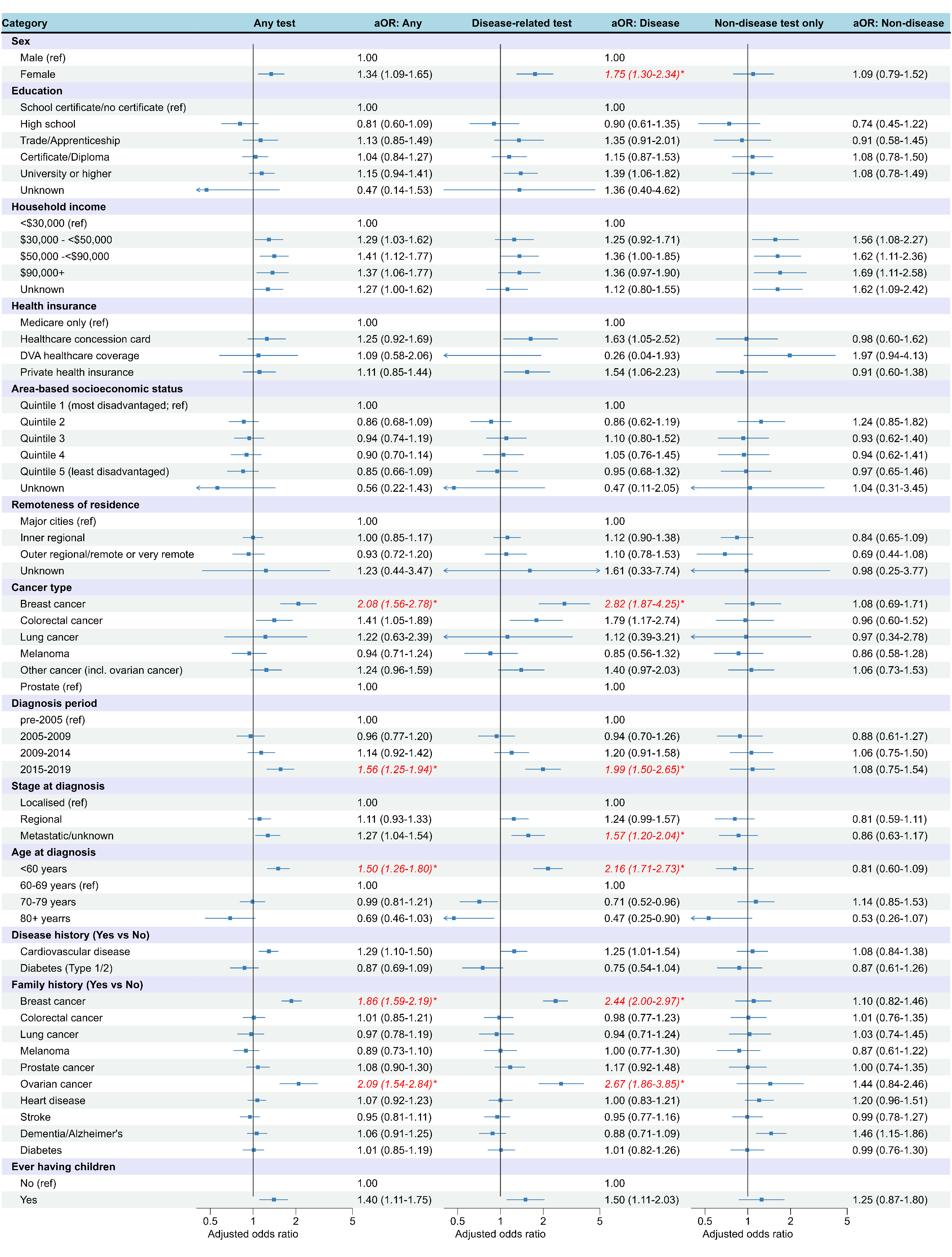


aOR: Odds ratio (OR)s adjusted for all characteristics shown here, alongside 95% confidence intervals in parentheses. Horizontal bars represent 95% confidence intervals; DVA: Department of Veterans' Affairs. Participants’ cancer type, year of diagnosis, age at diagnosis, and summary spread of disease at diagnosis were based on the first invasive cancer record.

* Associations significant at p<0.001 (Bonferroni-corrected threshold accounting for multiple testing).

**Note**: The reference category for both personal and family history of diseases was defined within each disease, i.e. estimates relate to participants with a specific disease compared to those without that specific disease, or to participants with family history of a specific disease to those without family history of that specific disease.

# 4. Association with income, without adjusting for educational attainment

Supplemental Fig. 3 Sensitivity analysis: associations between participants' characteristics and self-reported genetic testing, without adjustment for educational attainment (based on n=45,061 participants of the 45 and Up Study followed up in 2020 who were included in the analysis)


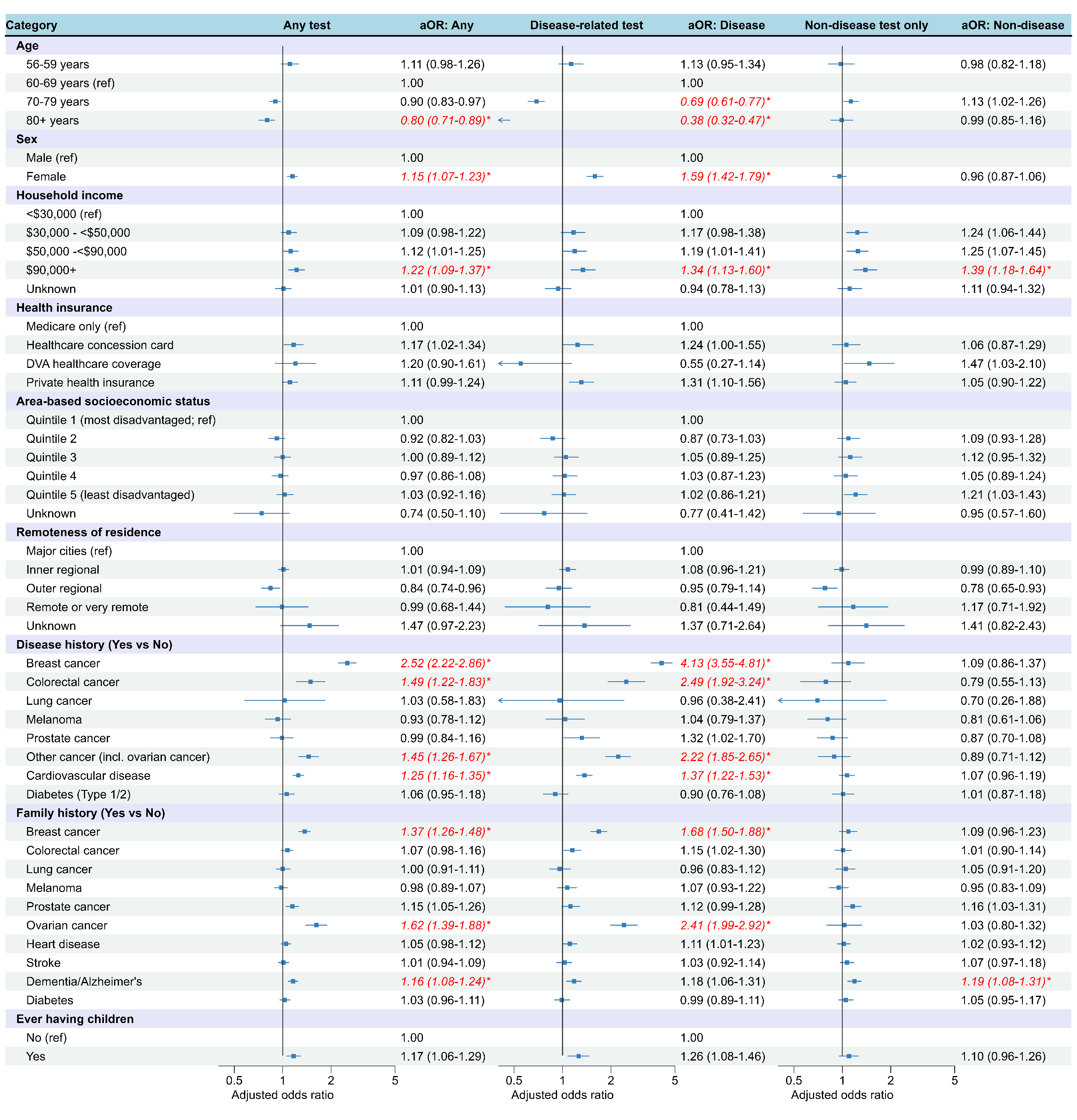


aOR: Odds ratio (OR)s adjusted for all characteristics shown here, alongside 95% confidence intervals in parentheses. Horizontal bars represent 95% confidence intervals; DVA: Department of Veterans' Affairs.

* Associations significant at p<0.001 (Bonferroni-corrected threshold accounting for multiple testing).

**Note**: The reference category for both personal and family history of diseases was defined within each disease, i.e. estimates relate to participants with a specific disease compared to those without that specific disease, or to participants with family history of a specific disease to those without family history of that specific disease.

Supplemental Fig. 4 Sensitivity analysis: associations between participants' characteristics and self-reported genetic testing among participants with a history of cancer, without adjustment for educational attainment

This analysis included 7,916 participants of the 45 and Up Study followed up in 2020 who were included in the analysis.


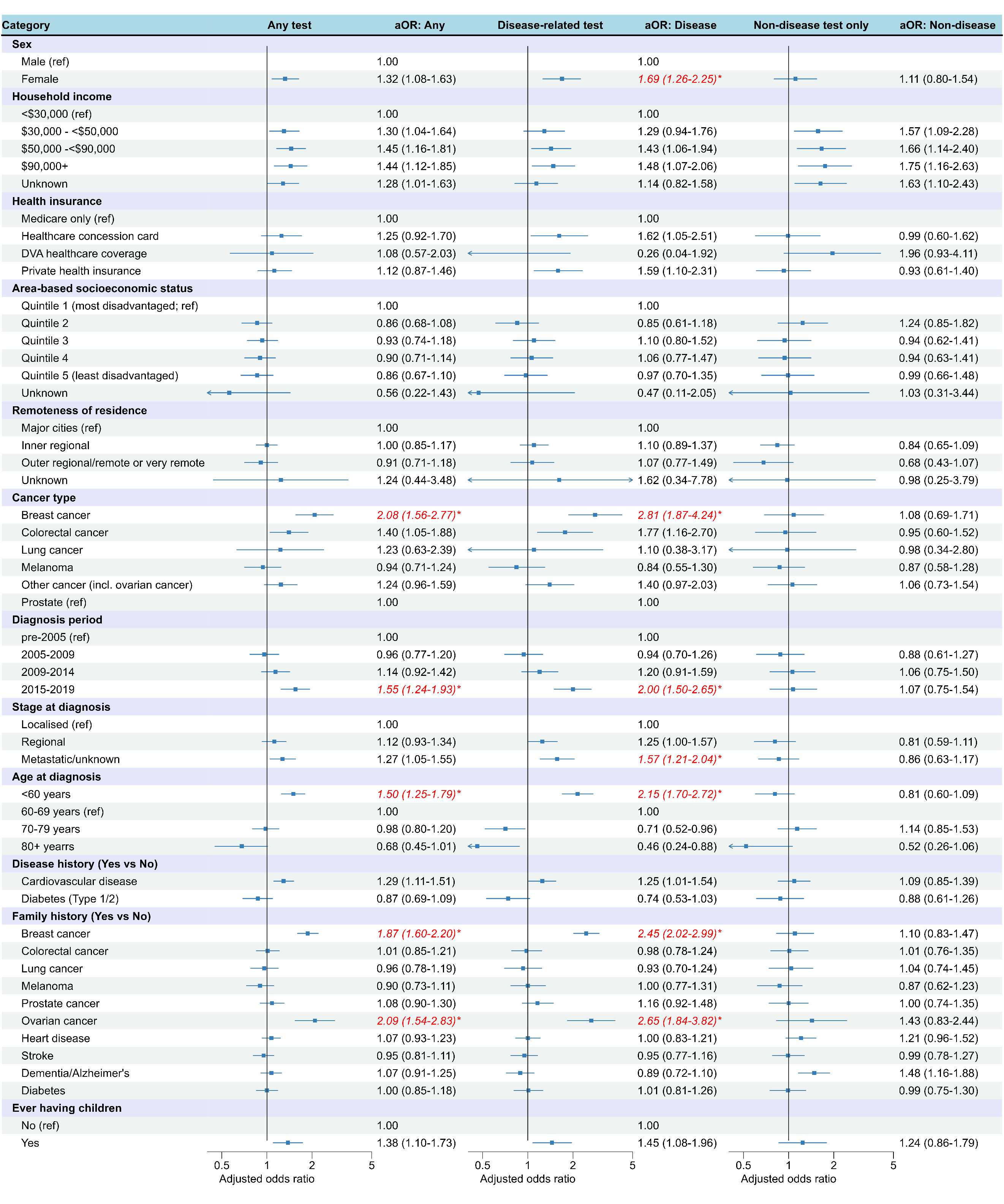


aOR: Odds ratio (OR)s adjusted for all characteristics shown here, alongside 95% confidence intervals in parentheses. Horizontal bars represent 95% confidence intervals; DVA: Department of Veterans' Affairs White or Gold Card. Participants’ cancer type, year of diagnosis, age at diagnosis, and summary spread of disease at diagnosis were based on the first invasive cancer record.

* Associations significant at p<0.001 (Bonferroni-corrected threshold accounting for multiple testing).

**Note**: The reference category for both personal and family history of diseases was defined within each disease, i.e. estimates relate to participants with a specific disease compared to those without that specific disease, or to participants with family history of a specific disease to those without family history of that specific disease.

# 5. Association with sex, after removing participants with personal or family history of cancer

Supplemental Fig. 5 Sensitivity analysis: association between participants’ characteristics and genetic testing among participants with no personal and no family history of cancer

This analysis included 17,530 participants of the 45 and Up Study followed up in 2020 who were included in the analysis.


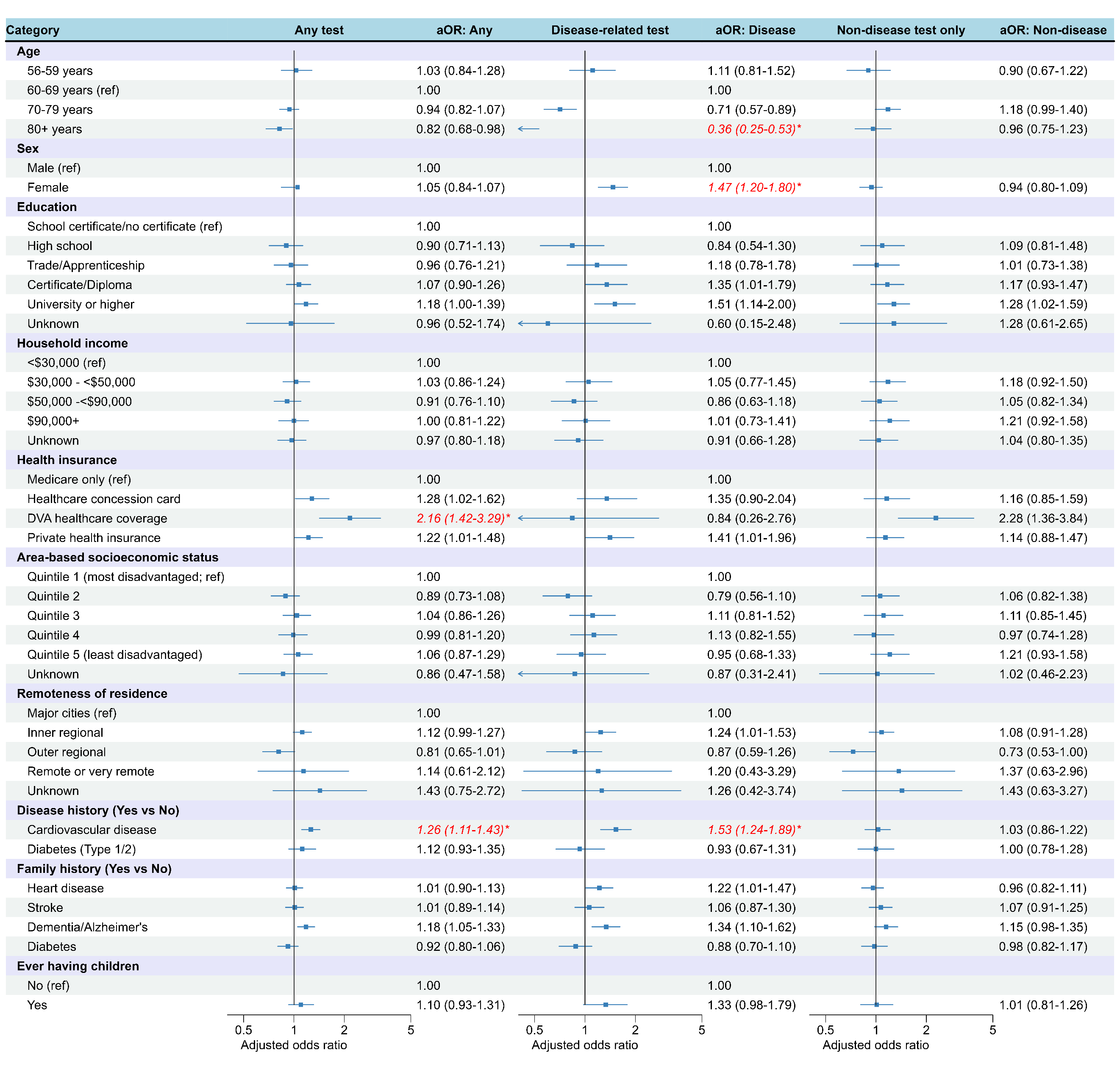


aOR: Odds ratio (OR)s adjusted for all characteristics shown here, alongside 95% confidence intervals in parentheses. Horizontal bars represent 95% confidence intervals; DVA: Department of Veterans' Affairs.

* Associations significant at p<0.001 (Bonferroni-corrected threshold accounting for multiple testing).

**Note**: The reference category for both personal and family history of diseases was defined within each disease, i.e. estimates relate to participants with a specific disease compared to those without that specific disease, or to participants with family history of a specific disease to those without family history of that specific disease.

# 6. Associations between genetic testing and participants’ characteristics, applying re-weighting to Australian Census data (people aged 55+ years)

As an exploratory analysis, we have now added a sensitivity analysis examining associations with genetic testing after re-weighting of the study data for selected sociodemographic characteristics to Australian Census data (people aged 55+ years). We completed separate analyses re-weighting for each of age, sex, education, household income, area-based socioeconomic status and remoteness/accessibility of area of residence (noting that re-weighting for a wide range of characteristics simultaneously is statistically complex and beyond the scope of the current study).

In general, the significant associations found in the main analysis (p<0.001) remained similar after re-weighting, with aOR estimates within the 95% CIs for aOR estimates from the main analyses (Supplemental Table 5). In a small number of exceptions as described in the following, weighting for education slightly changed the associations from the main analysis (aOR estimates outside for aOR estimates from the main analyses), but with no change in overall conclusions:

- Age: slightly stronger association between disease-related testing and age 70-79 years (*versus* 60-69 years), with aOR=0.70 (95% CI: 0.62-0.79) in the main analysis, and aOR=0.61 after weighting based on education.
- Sex: slightly stronger association between disease-related testing and female sex (*versus* male sex), with aOR=1.62 (95% CI: 1.44-1.82) in the main analysis, and aOR=1.88 after weighting based on education.
- Personal history of colorectal cancer: slightly stronger association between disease-related testing and personal history of colorectal cancer (*versus* no personal history of colorectal cancer), with aOR=2.50 (95% CI: 1.92-3.25) in the main analysis, and aOR=3.36 after weighting based on education.
- Personal history of CVD: slightly stronger associations of personal history of CVD (*versus* no personal history of CVD) with both any genetic testing and disease-related testing. We estimated aOR=1.25 [95% CI: 1.16-1.35] in the main analysis, and aOR=1.43 after weighting based on education for any genetic testing. For disease-related testing, the estimates were aOR=1.37 [95% CI: 1.22-1.53] in the main analysis, and aOR=1.57 after weighting based on education.
- Family history of ovarian cancer: slightly reduced associations between any genetic testing and family history of ovarian cancer (*versus* no family history of ovarian cancer), with aOR=1.62 (95% CI: 1.39-1.88) in the main analysis, and aOR=1.37 after weighting based on education.

# 7. Associations between genetic testing and participants’ characteristics, stratified by sex

When testing associations between genetic testing and participants’ characteristics separately for females and males, results were generally consistent with those of the main analysis (Supplemental Table 6). In particular, for associations with p<0.001 in the main analysis,

- association estimates stratified by sex were generally contained within the 95% CIs for estimates in the main analysis, and

- association estimates for males were within the 95% CIs for association estimates for females, and/or vice versa,

with a small number of exceptions as follows.

- Education: stronger association between university education and disease-related as well as non-disease-related genetic testing for males. For both disease-related and non-disease-related genetic testing, there was a stronger association in the analysis of males than of females (aOR estimate for males not contained within 95% CIs from analysis of females: disease-related testing aOR=2.25 [95%CI: 1.61-3.14] for males, aOR=1.31 [95% CI: 1.11-1.56] for females; non-disease-related testing: aOR=1.56 [95%CI: 1.24-1.97] for males, aOR=1.24 [95% CI: 1.04-1.47] for females). For both disease-related and non-disease-related genetic testing, there was also a stronger association in the analysis of males only than in the main analysis (aOR estimate for males not contained within 95% CIs from main analysis, which were aOR=1.50 [95%CI: 1.29-1.75] for disease-related testing and aOR=1.35 [95% CI: 1.17-1.54] for non-disease-related testing).
- Personal history of other cancer (including ovarian cancer): reduced association with any genetic testing for males. We detected an association with any genetic testing in the main analysis (aOR=1.45 [95%CI: 1.26-1.67]), with a stronger association observed in the analysis of females only (OR=1.74 [95%CI: 1.46-2.07], but no evidence for association in the analysis of males only (OR=1.09 [95%CI: 0.86-1.37]). [However, we note that for disease-related testing, there were significant associations in the main analysis as well as both sex-specific analyses, with the aOR estimate for females included in the 95%CI for the aOR for males.]
- Family history of breast cancer: evidence for association with any and disease-related testing for females but not for males. We detected an association with any genetic testing in the main analysis (aOR=1.36 [95%CI: 1.26-1.48]), with a similar association also observed in the analysis of females only (OR=1.59 [95%CI: 1.44-1.76]), but no evidence for association in the analysis of males only (OR=0.99 [95%CI: 0.85-1.16]). There was an association with disease-related genetic testing in the main analysis (OR=1.67 [95%CI: 1.49-1.88]), with a similar association also observed in the analysis of females only (OR=1.93 [95%CI: 1.69-2.20]), but no significant evidence for association in the analysis of males only (OR=1.12 [95%CI: 0.88-1.44]).

There were no associations with p<0.001 in the sex-stratified analyses that were not already identified in the main analysis.

# 8. Previous studies of genetic testing and associations with sociodemographic characteristics

Previous studies in Australia and the USA have identified several sociodemographic characteristics associated with access to genetic testing, including younger age, female sex (potentially due to gender differences in health awareness and attitudes toward preventative care), university education, existing knowledge of genomics, and socioeconomic advantage [9, 12, 13]. In particular, the Genioz study (2016-2017) in Australia found that genetic testing was more common for females (aOR=1.6 [95% CI:1.2-2.1] *versus* males), those currently studying/completed university (aOR=2.3 [95% CI:1.8-3.1] *versus* never studied at university) and participants working in genomics and/or life sciences (aOR=2.6 [95% CI:1.7-3.1] *versus* not working in genomics and/or life sciences) [9]. Furthermore, age was associated with a difference in the type of genetic test undertaken, with those in younger age groups more likely to undergo carrier testing, while older Australians were more likely to undertake testing for ancestry reasons [9, 12].

Availability of genetic testing has increased significantly since these studies were conducted, partly due to advancement in genomic technologies and the resulting lower costs, with as many people purchasing direct-to-consumer (DTC) tests in 2018 worldwide as in all previous years combined [14]. Thus, it is timely to complete a new investigation of associations between genetic testing and individuals’ sociodemographic characteristics. Notably, the large-scale data available in the 45 and Up Study allow the examination of a wider range of participants’ characteristics, including multiple different levels of highest educational qualification, as well as an area-based index of socioeconomic disadvantage.

# 9. Overlap in disease-related and non-disease-related genetic testing

In our study and elsewhere [9, 13], non-disease-related testing was reported by ~50% of participants who reported ever having any genetic testing. Our study also supports some overlap in testing purpose, with ~10% of participants who ever had any genetic testing reporting both disease-related and non-disease-related purposes.

Of potential relevance to this aspect is that data provided by direct-to-consumer genetic tests for non-disease-related purposes can be re-analysed by third-party companies to generate health-related insights without consultation of medical practitioners [15]. Interestingly, previous Australian research found that there is an expectation on the part of consumers to derive health-related information from non-disease-related testing, indicating an increasingly blurred boundary between the role of health professionals and patients [12]. As both direct-to-consumer genetic tests and third-party services are often performed outside of Australia with no Australian regulatory oversight [16], this presents challenges in assuring the quality of information received by consumers, and highlights the need for genomics education for healthcare professionals who may be subsequently consulted to help interpret results [17, 18].

# 10. Availability of disease-related DTC tests in Australia and considerations related to health disparities

We note that as of October 2024, several disease-related DTC tests are available in Australia, including tests to detect increased risk of cancer or cardiovascular disease due to rare pathogenic variants (for example, covering selected variants in 65 genes including *BRCA1, BRCA2, MLH1, MSH2, MSH6, PMS2* for cancer; and selected variants in 83 genes such as *MYBPC3, MYH7, FHL1* for cardiomyopathy and *ACVRL1, BMPR2, ENG, GDF2, SERPINC1, SMAD4* for high blood pressure) [19]. Detection of such pathogenic variants would then allow preventative actions to improve future health, such as enhanced cancer screening, or risk reducing medications for cancer or cardiovascular disease as relevant. However, these DTC genetic tests are currently (as of February 2025) not reimbursed through Medicare or private health insurance, and are thus only accessible to people who can cover the out-of-pocket costs (in the example above, currently ~950 AUD for cancer or cardiovascular traits each, or ~1,200 AUD for both combined). This has the potential to further widen existing disparities in health and premature mortality by socioeconomic status [20].

Another example of disease-related DTC tests available in Australia are reproductive carrier tests. While re-imbursement of tests through Medicare is limited to tests for a small number of conditions (cystic fibrosis, spinal muscular atrophy, and fragile X syndrome) as of October 2024, more comprehensive tests are available through health providers and as DTC tests [21]. For example, a more comprehensive DTC carrier test including >600 genes is currently available for ~950 AUD [19]. The out-of-pocket expense associated with such DTC tests limiting access could similarly contribute to widening health disparities across generations in the long term.

As an important general consideration for disease-related DTC tests, it should also be noted that only a limited number of genes and variants are covered, and false-positive results or misinterpretation of rare variants have been reported for some tests internationally [22]. Thus, it is also important to ensure that DTC tests do not lead to unnecessary health interventions nor provide false reassurance regarding disease risk.

#

# 11. References

1. Banks E, Redman S, Jorm L, Armstrong B, Bauman A, Beard J, et al. Cohort profile: the 45 and up study. International journal of epidemiology. 2008;37(5):941-7.

2. Bleicher K, Summerhayes R, Baynes S, Swarbrick M, Navin Cristina T, Luc H, et al. Cohort Profile Update: The 45 and Up Study. International journal of epidemiology. 2023;52(1):e92-e101.

3. Sax Institute. The 45 and Up Study. Wave 3 Data Book: Second Follow-Up, 2018-2020 2021 [Available from: <https://www.saxinstitute.org.au/wp-content/uploads/W3-databook-May2021.pdf>.

4. Emery JD, Reid G, Prevost AT, Ravine D, Walter FM. Development and validation of a family history screening questionnaire in Australian primary care. Annals of family medicine. 2014;12(3):241-9.

5. Houwink EJF, Hortensius OR, van Boven K, Sollie A, Numans ME. Genetics in primary care: validating a tool to pre-symptomatically assess common disease risk using an Australian questionnaire on family history. Clinical and translational medicine. 2019;8(1):17.

6. Australian Bureau of Statistics. An Introduction to Socio-Economic Indexes for Area (SEIFA). Canberra, Australia; 2006. Report No.: 2039.0. 2006.

7. Australian Bureau of Statistics. Australian Statistical Geography Standard: Remoteness Structure. Australia. 2011.

8. Bentley JP, Ford JB, Taylor LK, Irvine KA, Roberts CL. Investigating linkage rates among probabilistically linked birth and hospitalization records. BMC medical research methodology. 2012;12:149.

9. Savard J, Hickerton C, Tytherleigh R, Terrill B, Turbitt E, Newson AJ, et al. Australians' views and experience of personal genomic testing: survey findings from the Genioz study. European journal of human genetics : EJHG. 2019;27(5):711-20.

10. Australian Bureau of Statistics. Population: Census. ABS 2021 [Available from: <https://www.abs.gov.au/statistics/people/population/population-census/latest-release>.

11. Yap S, Luo Q, Wade S, Weber M, Banks E, Canfell K, et al. Raking of data from a large Australian cohort study improves generalisability of estimates of prevalence of health and behaviour characteristics and cancer incidence. BMC medical research methodology. 2022;22(1):140.

12. Metcalfe SA, Hickerton C, Savard J, Stackpoole E, Tytherleigh R, Tutty E, et al. Australians' perspectives on support around use of personal genomic testing: Findings from the Genioz study. European journal of medical genetics. 2019;62(5):290-9.

13. Swoboda CM, Wijayabahu AT, Fareed N. Attitudes towards and sociodemographic determinants of genetic test usage in the USA; data from the Health Information National Trend Survey, 2020. Journal of genetic counseling. 2023;32(1):57-67.

14. MIT Technology Review. More than 26 million people have taken an at-home ancestry test 2019 [Available from: <https://www.technologyreview.com/2019/02/11/103446/more-than-26-million-people-have-taken-an-at-home-ancestry-test/>.

15. Guerrini CJ, Wagner JK, Nelson SC, Javitt GH, McGuire AL. Who's on third? Regulation of third-party genetic interpretation services. Genetics in medicine : official journal of the American College of Medical Genetics. 2020;22(1):4-11.

16. Burns BL, Bilkey GA, Coles EP, Bowman FL, Beilby JP, Pachter NS, et al. Healthcare System Priorities for Successful Integration of Genomics: An Australian Focus. Frontiers in public health. 2019;7:41.

17. Cusack MB, Hickerton C, Nisselle A, McClaren B, Terrill B, Gaff C, et al. General practitioners' views on genomics, practice and education: A qualitative interview study. Australian journal of general practice. 2021;50(10):747-52.

18. Smit AK, Newson AJ, Keogh L, Best M, Dunlop K, Vuong K, et al. GP attitudes to and expectations for providing personal genomic risk information to the public: a qualitative study. BJGP open. 2019;3(1):bjgpopen18X101633.

19. eugene: Our Tests. [Available from: <https://eugenelabs.com>.

20. Adair T, Lopez AD. An egalitarian society? Widening inequalities in premature mortality from non-communicable diseases in Australia, 2006-16. International journal of epidemiology. 2021;50(3):783-96.

21. Australian Government. Department of Health and Aged Care. Medicare Benefits Schedule. Reproductive carrier testing for cystic fibrosis, spinal muscular atrophy and fragile X syndrome - Factsheet [updated 22/11/2023. Available from: <https://www.mbsonline.gov.au/internet/mbsonline/publishing.nsf/Content/0FAE1338D92EA3A3CA258A6F0001701A/$File/FS%20-%20Reproductive%20carrier%20testing%20for%20cystic%20fibrosis,%20spinal%20muscular%20atrophy%20and%20fragile%20X%20syndrome.pdf>.

22. Nolan JJ, Ormondroyd E. Direct-to-consumer genetic tests providing health risk information: A systematic review of consequences for consumers and health services. Clin Genet. 2023;104(1):3-21.
